# IN-HOSPITAL MORTALITY OF OLDER ADULT PATIENT WITH PROXIMAL FEMORAL FRACTURE TREATED SURGICALLY DURING THE COVID-19 PANDEMIC

**DOI:** 10.1590/1413-785220243204e278635

**Published:** 2024-10-07

**Authors:** Giuseppe Orsi Salazar, Guilherme Grisi Mouraria, Maurício Etchebehere, Rodrigo Gonçalves Pagnano

**Affiliations:** 1. Hospital das Clinicas da Universidade Estadual de Campinas (Unicamp), Unicamp, Campinas, SP, Brazil.

**Keywords:** Hospital mortality, Proximal Femoral Fractures, COVID-19 Serological Testing, Orthopedic Procedures, Mortalidade Hospitalar, Fraturas Proximais do Fêmur, Teste Sorológico para COVID-19, Procedimentos Ortopédicos

## Abstract

**Objectives:**

Evaluate the prevalence of hospital mortality in older adult patients with femoral fracture undergoing surgical treatment during the COVID-19 pandemic period, and to evaluate whether COVID-19 infection, clinical, and orthopedic factors interfered with mortality.

**Material and Methods:**

A retrospective study was conducted by reviewing medical records. Patients over 60 years of age with proximal femoral fracture undergoing surgical treatment were included. Overall mortality was calculated, as well as mortality whose primary or secondary cause was COVID-19 infection, to determine if infection influenced patient mortality. Clinical and orthopedic factors that interfered with mortality were evaluated. Categorical variables were compared using the Chi-square test or Fisher’s exact test. Both unpaired t-test (parametric variables) and Mann-Whitney test (non-parametric variables) were used. The Kaplan-Meier mortality curve was constructed.

**Conclusion:**

The mortality of older adult patients with femoral fracture undergoing surgical treatment during the COVID-19 pandemic was 4.2%. Male sex, older age, and those who underwent blood transfusion had higher mortality rates. COVID-infected patients had ten times more chance of death and died twice as fast as the non-infected population. ***Level of Evidence II, Retrospective Study.***

## RESUMO

**Objetivos:**

*Avaliar a mortalidade hospitalar de pacientes idosos com fratura de fêmur submetidos ao tratamento cirúrgico durante o período pandêmico de covid-19. Avaliar se a infecção pelo vírus do covid-19 e os fatores clínicos e ortopédicos interferiram na mortalidade.*

**Material e Métodos:**

*Realizou-se um estudo retrospectivo por levantamento de prontuários. Foram incluídos pacientes acima de 60 anos associados a fratura da extremidade proximal do fêmur e que submetidos a tratamento cirúrgico. Calculou-se a mortalidade geral e também aquela cuja causa principal ou secundária foi a infeção pelo covid-19 para determinar se essa influenciou na mortalidade dos pacientes. Foram avaliados se os fatores clínicos e ortopédicos interferiram na mortalidade e variáveis categóricas foram comparadas pelo teste de Qui-quadrado ou exato de Fisher, utilizando tanto o teste t não pareado (variáveis paramétricas) como o teste de Teste Mann-Whitney (variáveis não paramétricas). Por fim, construiu-se a curva de mortalidade de Kaplan-Meier.*

**Conclusão:**

*A taxa de mortalidade de pacientes idosos com fratura de fêmur submetidos ao tratamento cirúrgico durante a pandemia de Covid foi de 4,2%. Pacientes do sexo masculino, idosos e os que foram submetidos à transfusão sanguínea evoluíram com maior mortalidade. Pacientes infectados pelo Covid tiveram dez vezes mais chance de evoluir para óbito e de forma duas vezes mais rápida que a população não infectada. **Nível de Evidência II, Estudo Retrospectivo.***

## INTRODUCTION

 With the aging of the population, older adult’s health has become a growing concern, especially when linked to mortality associated with proximal femoral fractures and when combined with infectious diseases such as COVID-19. ^1^^-^^4^ Such conditions hinder clinical management and worsen the prognosis of patients. ^3^^,^^5^^,^^6^ In particular, the COVID-19 pandemic has caused significant disruption to health systems worldwide, particularly affecting vulnerable populations such as the older adults. ^3^^,^^6^^,^^7^

 In this context, this study seeks to better understand the relation between mortality and the coexistence of proximal femoral fracture and COVID-19 infection in older adult patients during hospitalization. Although numerous studies have shown increased mortality in patients with wfemoral fractures and co-infection with COVID-19, there are few Brazilian studies that have evaluated the mortality of this cohort of patients. In addition, the studies focused their evaluations on the first years of the pandemic. ^8^

Therefore, this study’s primary objective was to evaluate the prevalence of in-hospital mortality of older adult patients with femoral fractures undergoing surgical treatment during the COVID-19 pandemic. The secondary objective was to evaluate whether infection by the COVID-19 virus, clinical, and orthopedic factors (fracture location, type of surgical treatment employed, change in hemoglobin value) interfered with mortality.

## MATERIAL AND METHODS

A retrospective study was carried out based on medical records at a trauma referral hospital from January 2020 to December 2022.

Inclusion criteria were patients over 60 years of age who had fractures on the proximal extremity of the femur, who underwent surgical treatment, and who had all the clinical data as well as the death record or hospital discharge.

The exclusion criteria were patients who did not have all the clinical and orthopedic data in the medical records and patients who died before undergoing surgical treatment.

Demographic data, comorbidities (Hypertension, Diabetes, Hypothyroidism, Chronic Kidney Disease, Alzheimer’s, Depression, previous heart attack or previous stroke), preoperative hemoglobin value, and need for blood transfusion during hospitalization were evaluated.

Orthopedic factors evaluated were: the anatomical location of the femoral fracture (femoral neck, transtrochanteric, and subtrochanteric); types of surgical treatment used (osteosynthesis with nails, dynamic hip screw-plates, and cephalomedullary nails); variation of the pre- and postoperative hemoglobin value and, finally, the time between hospitalization and surgery.

Confirmation of COVID-19 infection was obtained by reverse transcription PCR (RT-PCR). The examination was routinely performed at the hospital during the pandemic and, therefore, symptomatic and non-symptomatic cases were diagnosed.

Overall mortality was calculated, as well as mortality in which the main or secondary cause was COVID-19 infection, to determine whether the infection influenced patient mortality. In addition, it was evaluated whether clinical and orthopedic factors interfered with patient mortality.

Categorical variables were tested by Fisher’s exact or Chi-square test, and the non-categorical variables by the Kolmogorov-Smirnov test.

Thus, to study these variables, both the unpaired t-test (parametric variables) and the Mann-Whitney test (non-parametric variables) were used.

The Kaplan-Meier mortality curve was designed. A significance level of p < 0.05 was considered. SPSS statistics program was used.

The study was approved by the Research Ethics Committee of UNICAMP under the CAAE No. 34076720.2.0000.5404.

## RESULTS

During the three years of research, 695 patients underwent surgical treatment to correct femoral fractures. However, 469 met all the inclusion criteria. There was a higher prevalence of women (64.4%). The mean age was 78.93+9.1 years. The most frequent comorbidity was hypertension and type II diabetes mellitus.

The most prevalent fracture was the transtrochanteric and the most used synthesis material was the short cephalomedullary nail.

 Demographic data of the sample are described in Table 1 . 

The mean time between hospitalization and surgery was 3.03+2.2 days. During the COVID-19 pandemic, seven patients were diagnosed with the disease by RT-PCR test, four of whom had respiratory complaints (symptomatic).

 The overall mortality rate during hospitalization was 4.2% (20 patients). Infection with the virus influenced mortality. Infected patients were ten times more likely to die than uninfected patients ( Table 2 ). 

 Male patients were twice as likely to die as female patients. In addition, older adult patients who underwent surgical treatment and died were, on average, seven years older than those who were discharged from the hospital. The presence of comorbidities ( Table 1 ) did not influence the mortality of patients, except for those who needed to receive blood transfusions ( Table 2 ). 

 The location of fracture, type of surgery (osteosynthesis or arthroplasty), difference in postoperative hemoglobin value, and the waiting time until the procedure did not influence mortality ( Table 3 ). 

**Figure 1.**
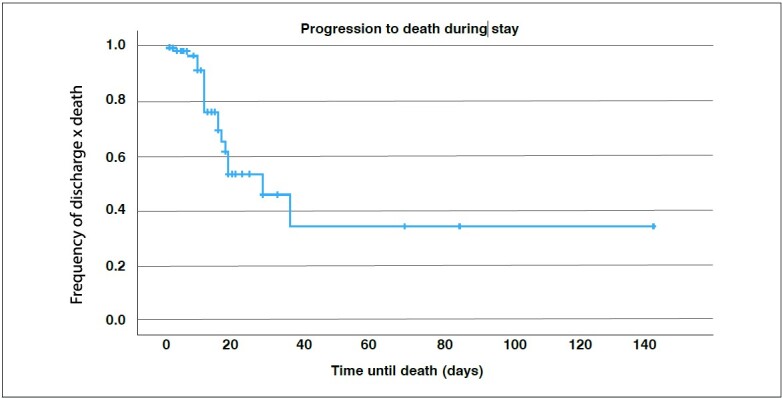
Kaplan Meier curve of in-hospital mortality of patients with femoral fractures

**Figure 2.**
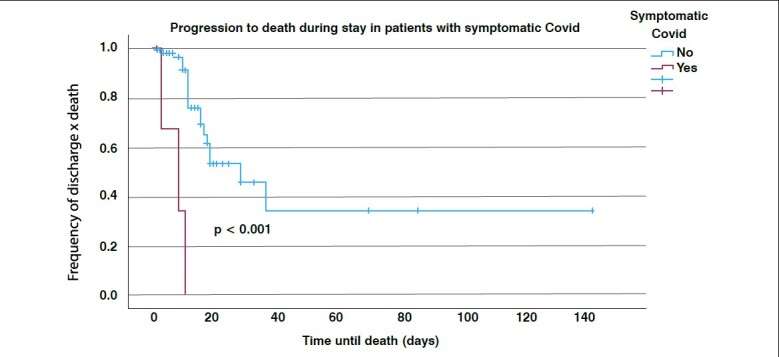
Kaplan Meier curve of in-hospital mortality of patients with femoral fracture associated or not with COVID-19 infection

 Most progressions to death occurred in the first twenty days of hospitalization ( Figure 1 ). Infection by COVID-19 determined a time acceleration for evolution to death ( Figure 2 ). 

**Table 1.** Demographic variables

Variable	Values
Age (mean + sd)	78.9 + 9.1
Sex [(N ^0^ (%)]	
Male	167 (35.6%)
Female	302 (64.4%)
Location of femoral fracture[N (%)]	
Transtrochanteric	259 (55.2%)
Neck	147 (31.3%)
Subtrochanteric	57 (12.1%)
Material Used in Surgery	
Short cephalodiaphyseal nail	242 (51.5%)
Dynamic hip screw-plates	23 (4.9%)
Long cephalodiaphyseal nail	57 (12.1%)
Bipolar arthroplasty	147 (31.5%)
Comorbidities [N ^0^ (%)]	
Hypertension	287 (61.1%)
Diabetes	136 (28.9%)
Hypothyroidism	52 (11%)
Chronic Kidney Disease	45 (9.5%)
Alzheimer’s disease	80 (17%)
Depression	45 (9.5%)
Previous heart attack	32 (6.8%)
Previous stroke	77 (16.4%)

**Table 2.** Influence of clinical variables on mortality

**Variable**	**Death (n)**	**Hospital Discharge (n)**	**P value**	**RR** *	**CI**
**COVID-19 infection (n)**					
Yes (7)	2	5	0.03 ^(a)^	0.11	0.23 - 0.53
No (462)	18	444		1.09	0.94 - 1.27
**Blood Transfusion (n)**					
Yes (78)	9	69	< 0.01 ^(b)^	0.34	0.20 - 0.58
No (391)	11	380		1.53	1.03 - 2.29
**Age (mean** + **sd)**	85.6 + 6.7	78.6 + 9.1	0.05 ^(c)^	-	-
**Sex (n)**					
Female (315)	9	306	0.04 ^(b)^	1.51	0.92 - 2.46
Male (154)	11	143		0.57	0.38 - 0.88

a Fisher’s exact test

* RR (Relative Risk Discharge/Death)

b Chi-squared

C t-test

**Table 3.** Influence of orthopedic variables on mortality

**Variable**	**Death (n)**	**Hospital Discharge (n)**	**P value**	**RR** *	**CI**
**Type of Surgery (n)**					
Osteosynthesis (322)	12	310	0.46 ^(a)^	-	-
Arthroplasty (147)	8	139		-	-
**Location of femoral fracture (n)**					
Transtrochanteric (261)	9	252	0.36 ^(a)^	-	-
Neck (149)	9	140	0.43 ^(a)^	-	-
Subtrochanteric (59)	2	57	0.81 ^(b)^		
**Hemoglobin drop (Median/min-max)**	0.1 (0-5)	0.1 (0-3)	0.15 ^(c)^	-	-
**Time until surgery (Mean + sd)**	3.25±2.6	3.02±2.2	0.61 ^(d)^	-	-

a Chi-square test

* RR (Relative Risk Discharge/Death)

b Fisher’s exact test

c Mann-Whitney test

d T-test

## DISCUSSION

In this retrospective observational study, the mortality of older adult patients with proximal femoral fractures and the clinic and orthopedic factors that could influence such mortality were evaluated.

 Older adult patients are known to be a vulnerable population and have numerous comorbidities. ^1^ Thus, during hospitalization for surgical treatment of the fracture, the presence of associated comorbidities is very frequent. ^4^ Our results also show this association, since at least 61% of the population had at least one comorbidity (hypertension). 

 The patients had a mean age of 78 years, with a higher prevalence among women. The result is similar to the international and national literature, which report greater prevalence in female patients, ^1^^,^^3^^,^^4^ with an average age of 79 years. ^4^^,^^9^ Several studies show that the risk of mortality from COVID-19 increases with age. ^8^^-^^10^ However, they reported a decrease in the incidence of fracture cases at the beginning of the pandemic, suggesting that there was a reduction in the demand for health services 11 ^,^ 12 due to the fear of contamination and the possibility of late sequelae related to COVID-19 infection. ^13^

 In our results, we observed a higher mortality among patients who underwent blood transfusion. Transfused patients were three times more likely to die than those who did not receive transfusion. However, patients who died had a similar decrease in hemoglobin values to those who were discharged from the hospital. The results restate the hypothesis that the higher mortality among transfused patients reflects a worse preoperative cynical condition than those that did not receive blood transfusion and is not related to the bleeding caused by the surgical procedure. In this context, we also observed that the type of surgery (arthroplasty or osteosynthesis) did not influence mortality. ^3^^,^^12^^,^^14^^,^^15^

 The prevalence of confirmed cases of COVID-19 in our study was 7 patients, which corresponded to approximately 1.5% of the total number of patients with femoral fractures during the period. Infection with the COVID-19 virus was devastating in this population cohort, as it increased the risk of death by 10 times. Meta-analysis with 23 studies 6 ^,^ 14 determined that the prevalence of COVID-19 was 13%, with a mortality rate of 35%, which corresponds to 7 times higher than those who did not have the associated infection. ^1^^,^^12^^,^^16^ Thus, although the prevalence of older adults with virus infection was lower in our study when compared to the literature, there was a high mortality rate, which may be related to worse general clinical condition of patients or a greater aggressiveness of the virus. 

 The highest mortality of patients occurred in the first 20 days of hospitalization, while, in those infected by COVID-19, it occurred earlier (mainly in the initial 10 days of hospitalization ( Figures 1 and 2 ). Meta-analysis with 20 studies comparing mortality between positive and negative COVID-19 patients, observed an increase in mortality rates ^17^ with a mean time to death of 30 days. ^12^ In addition, some studies point to a high rate of complications during hospitalization, such as sepsis, fluid and electrolyte imbalance. ^18^

 The mean waiting time until the surgical procedure was 3 days and did not influence patient mortality. Despite the literature pointing to an increase in waiting time for the procedure during the pandemic 2 ^,^ 5 ^,^ 18 ^-^ 20 , such increase did not occur in our survey, probably because patients with fractures were prioritized over those who were hospitalized for surgical treatment of elective pathologies. 

The study has some limitations, mainly related to the diagnosis of COVID-19 at the beginning of the pandemic, when diagnostic tests were still being implemented. Therefore, there is a possibility that some cases have not been diagnosed. Another important limitation is also related to a possible underreporting to the medical team of patients’ pre-existing comorbidities.

The study also presents some relevant aspects as it is the first national study that analyzed the mortality of patients with femoral fractures in older adult patients during the entire period of the COVID-19 pandemic and analyzed the influence of this infection on mortality. In addition, the study was conducted in a center specialized in the treatment of orthopedic trauma and that became a reference center for this disease as well during the pandemic.

## CONCLUSION

The mortality of older adult patients with femoral fractures undergoing surgical treatment during the COVID-19 pandemic was 4.2%. Older male patients who underwent blood transfusion had higher mortality.

Patients infected with COVID-19 were ten times more likely to die than those who were not infected. Therefore, the virus brought great morbidity to this cohort of patients, who died twice as quickly as the uninfected population.
